# Supplementary material for: Identification of photoperiod-induced specific miRNAs in the adrenal glands of Sunite sheep (Ovis aries)
Source: Front Vet Sci. 2022 Jul 22;9:888207. doi: 10.3389/fvets.2022.888207 (PMC9354845; doi:10.3389/fvets.2022.888207)
Supplement: Supplementary file 1 [file Data_Sheet_1.ZIP › Supplementary file/miRDeep2 file.zip!/miRDeep2 file/Know_Structure/SPLP42A2/oar-mir-16b.pdf]

miRBase precursor	: oar-mir-16b
Total read count	: 54896
oar-miR-16b read count	: 54855
remaining reads	: 41

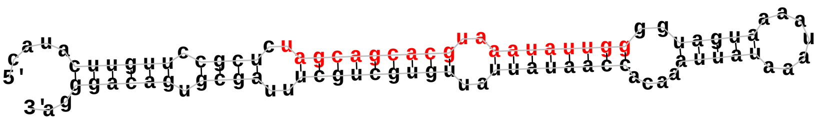

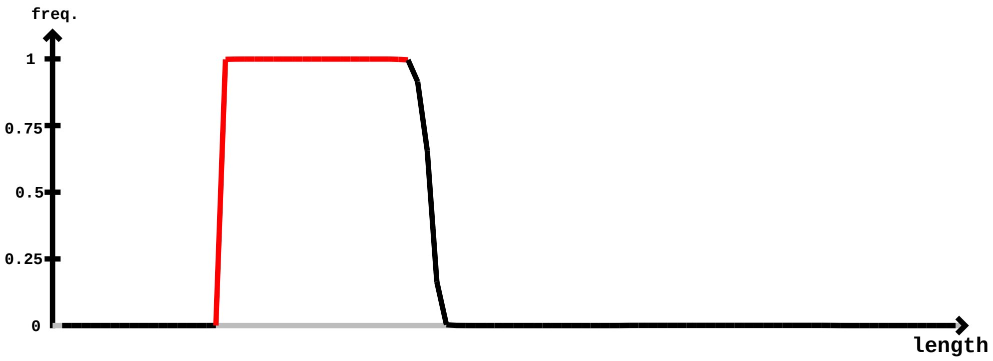

oar-miR-16b

5'- cauacuuguuccgcucuagcagcacguaaauauuggggguaguaaaauaaauauuaaacaccaauauuauugugcugcuuuagcgugacaggga -3' exp

....((((((.((((..(((((((((..((((((((..(((((.......)))))....))))))))..)))))))))..)))).))))))..	reads	mm	sample
..............ucuagcagcacguaaauauugg...	1	0	seq
...............cuagcagcacguaaauauugg...	3	0	seq
...............cuagcagcacguaaauauuggg..	1	0	seq
...............cuagcagcacguaaauauuggggu..	7	0	seq
................uagcagcacguaaauau..	2	0	seq
................uagcagcacguaaauauu...	60	0	seq
................uagcagcacguaaauauug..	105	0	seq
................uagcagcacguaaauauugg...	4469	0	seq
................uagcagcacguaaauauuggA..	12239	1	seq
................uagcagcacguaaauauuggg..	1914	0	seq
................uagcagcacguaaauauugggg...	2295	0	seq
................uagcagcacguaaauauuggAg...	24705	1	seq
................uagcagcacguaaauauuggggu..	2882	0	seq
................uagcagcacguaaauauuggAgu..	5948	1	seq
................uagcagcacguaaauauuggggua...	147	0	seq
................uagcagcacguaaauauugggguag..	15	0	seq
................uagcagcacguaaauauugggguagu...	5	0	seq
.................agcagcacguaaauauugg...	2	0	seq
.................agcagcacguaaauauuggg..	1	0	seq
.................agcagcacguaaauauuggA..	8	1	seq
.................agcagcacguaaauauuggAg...	21	1	seq
.................agcagcacguaaauauugggg...	2	0	seq
.................agcagcacguaaauauuggggu..	5	0	seq
.................agcagcacguaaauauuggAgu..	6	1	seq
.................agcagcacguaaauauuggggua...	1	0	seq
..................gcagcacguaaauauugg...	1	0	seq
..................gcagcacguaaauauuggA..	2	1	seq
..................gcagcacguaaauauuggAg...	9	1	seq
...................cagcacguaaauauugg...	1	0	seq
...................cagcacguaaauauuggA..	2	1	seq
...................cagcacguaaauauuggAg...	1	1	seq
....caccaauauuauugugcugcuu..............	2	0	seq
....caccaauauuauugugcugcuuu.............	2	0	seq
....accaauauuauugugcugcuu..............	5	0	seq
....accaauauuauugugcugcuuu.............	19	0	seq

oar-miR-16b

```
cauacuuguuccgcucuagcagcacguaaauauugggguaguaaaauaaauauuaaacaccaauauuauugugcugcuuuagcgugacaggga
..........................................................accaauauuauugugcugcuuua............    7          0          seq
...........................................................ccaauauuauugugcugcuuua............    1          0          seq
```
